# Supplementary material for: Characterization of biofilm production in different strains of Acinetobacter baumannii and the effects of chemical compounds on biofilm formation
Source: PeerJ. 2020 May 28;8:e9020. doi: 10.7717/peerj.9020 (PMC7261477; doi:10.7717/peerj.9020)
Supplement: Table S1 [file peerj-08-9020-s001.docx]

Table S1

|  | Strains | SAM | TZP | CZ | CMZ | CTX | CAZ | FEP | IPM | MEM | AN | GM | CIP | LVX | SXT | TGC |
| --- | --- | --- | --- | --- | --- | --- | --- | --- | --- | --- | --- | --- | --- | --- | --- | --- |
| Strong Biofilm Producers | VGH2 | R(≧32) | R(≧128) | R(≧64) | R(≧64) | R(≧64) | R(≧64) | R(32) | I(8) | R(≧16) | R(≧64) | R(≧16) | R(≧4) | R(≧8) | R(≧320) | R(8) |
|  | 17978 | S(≦2) | S(≦4) | R(≧64) | R(32) | S(8) | S(2) | S(≦1) | S(≦0.25) | S(≦0.25) | S(≦2) | S(≦1) | S(≦0.25) | S(≦0.12) | S(40) | S(≦2) |
|  | ABhl1 | I(16) | R(≧128) | R(≧64) | R(≧64) | R(≧64) | R(≧64) | R(32) | R(≧16) | R(≧16) | S(4) | R(≧16) | R(≧4) | R(≧8) | R(≧320) | R(8) |
|  | CM4 | I(16) | R(≧128) | R(≧64) | R(≧64) | R(≧64) | R(≧64) | R(≧64) | R(≧16) | R(≧16) | S(≦2) | R(≧16) | R(≧4) | R(≧8) | S(≦20) | S(≦2) |
|  | CM1 | R(≧32) | R(≧128) | R(≧64) | R(≧64) | R(≧64) | R(≧64) | R(≧64) | R(≧16) | R(≧16) | S(≦2) | S(≦1) | R(≧4) | R(≧8) | S(≦20) | S(≦2) |
|  | HC2 | R(≧32) | R(≧128) | R(≧64) | R(≧64) | R(≧64) | R(≧64) | R(≧64) | R(≧16) | R(≧16) | S(4) | R(≧16) | R(≧4) | R(≧8) | R(160) | S(≦2) |
|  | HC3 | R(≧32) | R(≧128) | R(≧64) | R(≧64) | R(≧64) | R(≧64) | R(32) | R(≧16) | R(≧16) | R(≧64) | R(≧16) | R(≧4) | R(≧8) | R(≧320) | S(≦2) |
|  | CM2 | S(4) | R(≧128) | R(≧64) | R(≧64) | R(≧64) | R(≧64) | I(16) | R(≧16) | R(≧16) | S(≦2) | R(≧16) | R(≧4) | R(≧8) | R(≧320) | S(≦2) |
|  | HC1 | I(16) | R(≧128) | R(≧64) | R(≧64) | R(≧64) | R(≧64) | R(≧64) | R(≧16) | R(≧16) | S(4) | R(≧16) | R(≧4) | I(4) | S(≦20) | S(≦2) |
|  | HC4 | R(≧32) | R(≧128) | R(≧64) | R(≧64) | R(≧64) | R(≧64) | R(≧64) | R(≧16) | R(≧16) | R(≧64) | R(≧16) | R(≧4) | R(≧8) | R(≧320) | S(≦2) |
|  | CT14 | R(≧32) | R(≧128) | R(≧64) | R(≧64) | R(≧64) | R(≧64) | R(≧64) | R(≧16) | R(≧16) | S(8) | R(≧16) | R(≧4) | R(≧8) | R(160) | S(≦2) |
|  | CM3 | I(16) | R(≧128) | R(≧64) | R(≧64) | R(≧64) | R(≧64) | R(32) | R(≧16) | R(≧16) | S(≦2) | R(≧16) | R(≧4) | R(≧8) | R(≧320) | S(≦2) |
|  | VGH4 | R(≧32) | R(≧128) | R(≧64) | R(≧64) | R(≧64) | R(≧64) | R(≧64) | R(≧16) | R(≧16) | S(≦2) | R(≧16) | R(≧4) | R(≧8) | R(≧320) | R(8) |
|  | VGH5 | R(≧32) | R(≧128) | R(≧64) | R(≧64) | R(≧64) | R(≧64) | R(≧64) | R(≧16) | R(≧16) | S(≦2) | R(≧16) | R(≧4) | R(≧8) | R(≧320) | S(≦2) |
|  | CT12 | R(≧32) | R(≧128) | R(≧64) | R(≧64) | R(≧64) | R(≧64) | R(≧64) | R(≧16) | R(≧16) | R(8) | R(≧16) | R(≧4) | R(≧8) | R(160) | S(≦2) |
|  | VGH7 | R(≧32) | R(≧128) | R(≧64) | R(≧64) | R(≧64) | R(≧64) | R(32) | R(≧16) | R(≧16) | R(≧64) | R(≧16) | R(≧4) | R(≧8) | R(≧320) | R(≧8) |
|  | CT13 | I(16) | R(≧128) | R(≧64) | R(≧64) | R(≧64) | R(≧64) | R(32) | S(1) | S(1) | S(≦2) | S(≦1) | R(≧4) | R(≧8) | R(≧320) | S(≦2) |
|  | VGH6 | I(16) | R(≧128) | R(≧64) | R(≧64) | R(≧64) | R(≧64) | R(≧64) | R(≧16) | R(≧16) | S(8) | R(≧16) | R(≧4) | R(≧8) | S(≦20) | R(≧8) |
|  | VGH3 | I(16) | R(≧128) | R(≧64) | R(≧64) | R(≧64) | R(≧64) | R(32) | I(8) | R(≧16) | S(≦2) | R(≧16) | R(≧4) | R(≧8) | R(≧320) | R(8) |
|  | HC5 | R(≧32) | R(≧128) | R(≧64) | R(≧64) | R(≧64) | R(≧64) | R(32) | R(≧16) | R(≧16) | S(4) | R(≧16) | R(≧4) | R(≧8) | R(160) | S(≦2) |
| Moderate Biofilm Producers | VGH1 | R(≧32) | R(≧128) | R(≧64) | R(≧64) | R(≧64) | R(≧64) | R(≧64) | R(≧16) | R(≧16) | S(4) | R(≧16) | R(≧4) | R(≧8) | R(≧320) | S(≦2) |
|  | CT11 | S(4) | R(≧128) | R(≧64) | R(≧64) | R(≧64) | R(≧64) | I(16) | S(1) | S(0.5) | S(4) | R(≧16) | R(≧4) | R(≧8) | R(160) | S(≦2) |
